# Ebola: Perspectives from a Nurse and Patient

**DOI:** 10.4269/ajtmh.14-0762

**Published:** 2015-02-04

**Authors:** Will Pooley

**Affiliations:** King's College Sierra Leone Partnership, Freetown, Sierra Leone

In July of 2014, I was working as a nurse for a small non-governmental organization in the Sierra Leone capital, Freetown, as the Ebola virus disease (EVD) epidemic grew in the east of the country. The reason that I had decided to study tropical nursing after completing my basic registered nurse training was because I wanted to apply my professional skills where they were most needed. Thus, it was not difficult for me to decide to work in the eastern city of Kenema, which at the time, was at the heart of the epidemic and the site of one of two Ebola treatment units (ETUs) in Sierra Leone. The situation in Kenema was tense; the regular nurses at the ETU were on strike over issues related to pay, but also, understandably, there was a strong undercurrent of fear, because they had already seen quite a few coworkers contract EVD, many fatally. The hospital management and expatriate World Health Organization (WHO) doctors were struggling desperately to keep the staffing up to something close to a safe and effective level.

I had no real plan of what to do on arrival in Kenema but eventually, was put in touch with the Kenema Government Hospital deputy matron, who immediately and enthusiastically accepted my offer to volunteer in the ETU. The next morning, I joined about 50 other nurses and community health officers—all potential new recruits, all Sierra Leoneans—at an introductory training session held by one of the WHO physicians. That afternoon, I met the small group of local staff and expatriate WHO clinicians working to provide and organize EVD care in Kenema. One of the WHO doctors showed me how to don and doff the personal protective equipment, and we did a walkthrough of the ETU. The next day, I started work, one of the only nurses in the facility. Clearly, not many of the new recruits had heeded the call. The hours were simple: all day, every day.

Conditions in the ETU were grim. It was comprised of three wards: a smaller one designated for suspect cases and two larger ones for those with confirmed EVD, altogether totaling around 70 beds. The layout, which ordinarily would result from careful planning to implement strict infection prevention and control measures, was entirely improvised. The largest ward of around 30 beds was simply one large room rendered from plastic sheeting covering a wooden frame with a laminate roof. There was much death, suffering, and neglect, with patients asking for simple things, like food and water. The high patient load, insufficient staff, and lack of resources pre-disposed the ETU to experiencing perhaps the highest rate of healthcare worker infection ever seen in an EVD epidemic. In my first few weeks working in the ETU, a healthcare worker died of EVD every few days. We all knew that the situation was far from ideal and far from safe, but what could we do? The patients with EVD were there, and someone had to take care of them. Sending them back home to their communities was not an option.

I worked in the ETU for 6 weeks before I got infected, which considering how prevalent healthcare worker infections were and the hours that I was working, was a pretty good run. Then, the mother of an 18-month-old boy died of confirmed EVD in the ETU. The baby appeared healthy, however, and tested RT-PCR–negative, much to the delight of the nursing team. These situations with possibly exposed orphaned children are always challenging. Is the child infected or not? Placed in the ETU, the child risks infection, and nutritional needs are hard to meet. However, sending the possibly infected child home risks transmission to family members if he falls sick. We decided to care for him in the nurses' area adjacent to the high-risk area of the ETU. We cared for and played with him wearing only minimal protective equipment, and he was fine for a few days; however, looking back on it, he might have had a few loose stools (but don't all babies?), and of course, we were changing his diapers. Babies cannot tell you how they feel, and therefore, it is hard to say the time of the onset of true symptoms. But then he developed a high fever. We isolated and retested him. The result, considerably delayed by a lost sample, came back positive. I fell sick 2 days after the baby was moved into the ETU, where he ultimately died.

Everyone working in EVD response pays close attention to what their bodies are telling them, but a certain amount of denial is inevitable. Therefore, when I awoke after a feverish night with a headache, myalgia, and an unfamiliar feeling of being really off, I thought that I would see how the day went before approaching a colleague. By midday, I felt worse, and it became clear that I had to get tested. I went to the WHO office, where my blood was taken. I remember a certain amount of dark humor surrounding the succession of events; I was given the diagnosis at home that evening by a WHO doctor in full personal protective equipment. After breaking the news in the gentlest way possible, he asked for my passport number, because apparently, the work on my medivac had already begun. During 36 hours of home isolation, I had one period of emotional upset precipitated by attempts to write a letter to my family to be read in the event of my death. Otherwise, my isolation was a matter of practicalities, with constant phone calls and many visitors making arrangements for the medivac. There was even a fair bit of laughter drawn from the surreal quality of the situation; when I called back to the United Kingdom in an attempt to inform my family of the news, I caught them during my cousin's wedding, for which my extended family had gathered on a blissful summer's afternoon in the English countryside. I opted for passing on congratulations to my cousin and the bride and chatting casually with the family. I delayed sharing the fact that I had EVD until a more appropriate moment later that day.

From my perspective, the medivac went ahead without a hitch, although I have since spoken to a number of people who worked frantically behind the scenes to organize my repatriation. During the 5-hour journey to the airfield, I concentrated on not developing vomiting or diarrhea—more dangerous symptoms in which virus is shed into the environment—that could make people rethink the evacuation plans. During the flight, I was placed in a mobile isolator tent, intermittently febrile and uncomfortable. Back in the United Kingdom, I was admitted to the Royal Free Hospital in west London, which was an education in world class care. Droves of excited infectious diseases consultants came to evaluate me. I received the experimental therapy ZMapp and made a very quick recovery. Before this experience, I had thought that, for patients, medical skill and outcomes would be the dominant themes in the perception of care. However, my memories of the Royal Free Hospital are not of the undeniable skill of the physicians or the details of my medical treatment but rather, are of the warmth, compassion, patience, and kindness shown by the staff to me and my family. All those working in healthcare should remember that kindness is the most essential quality in the healthcare worker.

After my recovery, I returned to Sierra Leone to continue working in the struggle against EVD. In the United Kingdom, I had often been asked, “How do you deal with witnessing all the death and suffering in an ETU?” But interestingly, this question has never been posed to me by a healthcare worker or anyone else who lives and works here in west Africa, and it always leaves me stumped. People in west Africa deal well when presented with extreme healthcare environments; I have worked with countless colleagues to provide patient care for EVD and have never seen one crack or crumble into an emotional wreck. People working in first-world healthcare as well as resource-poor settings will equally know the farthest reaches of human suffering. After all, there is no more pitiable condition than the demented patient in the grip of endless terrors. We all, as healthcare workers, know the worst of what it is to be alive, and here, in west Africa most other people do too. That question that I am often asked in the United Kingdom exposes a degree of ignorance in the first world, where human experience has become so narrowed by modern convenience that knowledge of the least pleasant bits of life and death is feared like contagion itself.

